# The development of a suitable training model for students with disabilities at a training institution in South Africa

**DOI:** 10.4102/ajod.v11i0.949

**Published:** 2022-12-09

**Authors:** Johanna C. Janse van Rensburg-Welling, Jean E. Mitchell

**Affiliations:** 1 National Institution for Development and Training NPC, Worcester, South Africa; 2 Department of Post Graduate Studies, Faculty of Education, The Da Vinci Institute for Technology Management, Johannesburg, South Africa; 3 Department of Research, National Institution for Development and Training, Worcester, South Africa

**Keywords:** training, assessment, training models, student-centred, accommodation, South Africa

## Abstract

**Background:**

The large South African population of people with disabilities presents unique challenges for training organisations because there is no training model that accommodates the demands of all disabilities. The site of the research was a private, nonprofit training institution with disability-friendly infrastructure which did not adequately prepare students for employment.

**Objectives:**

The intention was to interrogate training models used at the institution, from the perspectives of students, facilitators and prospective employers. As there was no suitable assessment instrument, one that was fit for purpose was developed. The Adaptable Component-based Assessment Model (ACA Model) was the result.

**Method:**

A case study using mixed methods was employed. The interpretive research paradigm allowed for purposive sampling. This article reports on the qualitative first phase of the study. The ACA Model was developed, and iterative applications provided information about areas that needed improvement before the second phase was initiated.

**Results:**

The results all indicated that the existing programmes needed to be evaluated with the view for improvement. Various training models can be used to train students with disabilities, but they need to be assessed to ensure that they are integrated, holistic and student centred. Because different accommodations need to be taken into account for various disabilities, the ideal assessment model needs to be adaptable.

**Conclusion:**

The ACA Model is an appropriate assessment model as it is based on individual learner affordances, workplace affordances, the holistic development of students and workplace absorption.

**Contribution:**

The research contributes to knowledge and practice as the resultant ACA Model can be used to the benefit of students and education institutions. The model can be tailored to the needs of all groups of students, especially those with disabilities.

## Introduction

Globally, persons with disabilities have limited access to education, training and employment (Maart, Amosun & Jelsma 2019). This is an important issue because it influences the achievement of a number of the United Nations Sustainable Development Goals that aim to eradicate poverty (Vornholt et al. 2018). The Bill of Rights, enshrined in the South African Constitution (RSA 1996) and the National Skills Development Strategy (RSA 2016) are just two of many strategies and laws that govern education, training and employment in South Africa. Unfortunately, the Acts, regulations and codes of best practice have not yet been implemented extensively enough for their impact to be measured (Human Rights Watch 2019, 2020). In South Africa, as in many other developing countries, being disabled increases a person’s chance of being un- or under-educated, unemployed and extremely poor (RSA 2018, 2019).

While it is accepted that disabilities are very complex and worthy of research (Bolt 2015), the purpose of the research reported here was to create a way to analyse training programmes offered by training institutions that cater for students with various disabilities. The information gained from this investigation informed the development of a model to analyse programmes offered not only at the target institution but also at other institutions that offer training programmes to people with disabilities. All programmes should meet the vision and mission of a training institution. At the same time, they should meet the complex demands and costs of providing education to people with disabilities effectively and efficiently while ensuring equal opportunities, economic growth and innovation (UNESCO 2020). Implementing training assessment or evaluation models are ways to achieve these goals.

The site of the research was a private, non-profit institution registered as a Public Benefit Organisation; its disability-friendly infrastructure made it ideal to train and support students with disabilities. It also acknowledged and respected disability culture (Bedoin 2019). Most enrolled students came from needy households who depended on social grants and could not afford to pay fees; thus, the institution needed to operate in an educationally and financially responsible manner, without succumbing to a culture of McDonaldisation where efficiency, calculability, predictability and, particularly, standardisation (Ritzer 2013) are the benchmarks.

At the time of the research, the programmes offered by the Training Department at the institution did not serve all the education and employment needs of students and were not financially sustainable. The actual needs of industries for which students were trained were not known. It also became evident that the training model in use did not adequately prepare students to enter employment in nondisabled situations in the 21st century.

It was imperative to interrogate training models used at the institution and to find a way to provide training that would best suit students with disabilities, the Training Department and prospective employers. The main research question asked how the Training Department could change the way in which it offered programmes to provide training that would lead to students gaining meaningful access to workplaces. Unfortunately, there was no suitable evaluation tool to assess the programmes. In this article, we focus on an analysing instrument that was developed for this purpose.

## Literature review

In South Africa, there are many people with disabilities who have limited access to education and training. Their chances of access to employment are lower than those of nondisabled people (Human Rights Watch 2020; Rathmann 2019). Each disability presents a unique challenge to the person affected and to those who provide appropriate training and support (Camarata et al. 2018).

Achieving and sustaining post-school education of high quality for students with disabilities needs the input from many stakeholders, including education institutions, students and prospective employers. Curricula should not reflect the homogenisation suggested by McDonaldisation (Crossman 2021) but should be responsive to social contexts (Lubbe, Wolvaardt & Turner 2020). The focus should move from learning ‘for work’, to ‘learning at work’ and even ‘learning through work’ (Garwe 2020:193).

Businesses that employ persons with disabilities require them to have the necessary skills and competencies to do their work (Garwe 2020). In addition, employers usually expect employees to have intangible, meta competencies like being able to self-regulate, being flexible and able to adapt to various work and social environments, anticipating and learning (Heery & Noon 2017). Yet despite advances in diversity and inclusion practices in the workplace, the entry and progression of people with disabilities in the workforce remain problematic and employers have negative opinions of their work-related abilities (Bonaccio et al. 2020). Indeed, Vornholt et al. (2018) argue that most employers hold unsupported stereotypical beliefs.

It seems as if employers often lack the values that lead to respecting democratic, professional, ethical and people values. Thus, they fail to build respectful, diverse and inclusive workplaces where they hold themselves and their employees accountable for their actions (Western Cape Government 2020). It seems that when employers do employ persons with disabilities, they tend to focus on providing physical and structural accommodations or affordances but ignore the emotional and psychological well-being of their employees (Vornholt et al. 2018). In addition, it seems as if prospective employers are often not considered in training programmes; thus, the needs of future employers are unknown.

The challenge in the present research was to find an instrument that could serve as a benchmark by which to analyse and assess the success of the training models used at the institution so that informed decisions would guide future programme adjustments. The instrument needed to provide consistent information and be applicable to future programme development. In addition, the instrument had to include all the components needed in the training and work placement competencies while providing adequate accommodations for students with disabilities. The benchmark instrument also had to comply with the laws of the land, offer meaningful education, prepare students for the world of work and prepare workplaces to welcome and accommodate students and employees with disabilities (Lubbe et al. 2020).

When reference is made to affordances or accommodations for disabled employees in the workplace, it usually means modifications that have been made to adapt to the special needs of an individual or group. It can also refer to adaptations made in workplaces to afford employees opportunities for learning (Dokumaci 2020). The aim of education for employment of students with disabilities should include a practical approach to a comprehensive learning system where physical, social, emotional, intellectual and spiritual growth are taken into account. Students should also be encouraged to be reflective learners. In other words, they should be encouraged to review their own learning in relation to their own lives and work environments to make meaning of the experience (Brockbank, McGill & Beech 2017).

Training ecosystems consist of the people, procedures and instruments used by an organisation to develop and support learning of theory and subject content and performance in the workplace (Benedicks 2018). Training ecosystems also allow participants (facilitators and students) to select the most appropriate technologies to help them accommodate their individual disabilities (Carlson 2019).

At the same time, evaluation of formal, informal, work-based and performance-supported training and post-training is necessary for the continued success of an institution. Such evaluation can determine the effectiveness of various components of the training and development programmes on offer (Alsalamah & Callinan 2021). Such an ecosystem supports learning and performance through social learning and knowledge sharing, performance support and repeated reinforcement of training and learning (Benedicks 2018), in fact, all the important elements in the education of students with disabilities.

Models that are meant to evaluate training are frameworks that provide a system or method to analyse training. They tend to focus on the success of the training and learning that has taken place, applicability to available employment, impact of the training, return on investment and improvements that can be made (Deller 2021). While there are several training models used in formal and informal education and training settings (Aquino 2016; Deller 2021), none of them was found to be suitable to address the complexities of the education of disabled persons at the institution or within the South African Education and Training System. Thus, other education and industry models were consulted.

The New World Kirkpatrick model was regarded as the most suitable for this research and was selected to guide the development of components against which the existing training could be assessed (Alsalamah & Callinan 2021). This decision was made after other models and theories had been interrogated. The researchers did not find the Context, Input, Reaction, Outcome (CIRO) Model of Warr, Bird and Rackham to be useful in this instance because it focuses on assessing the training of businesses managers and not training of unemployed people with disabilities (Harapa 2021). In the same way, the success case method (SCM) of Brinkerhoff (2005) was regarded as inappropriate because it was too wide for the purpose of the research. While the Phillips model is similar to that of Kirkpatrick, it was also regarded as unsuitable because it includes a cost–benefit aspect that was not necessary under the circumstances.

The four levels of criteria in Kirkpatrick’s model, namely reaction, learning, behaviour and results, were regarded as a valid starting point. Thus, the model enabled the focus to move from assessment of training to assessment of results achieved and the relevance the training had to individual workplaces.

## Research method

Data used for this article are based on a larger study conducted. The study was interpretive and used mixed methods within a case study design. This approach was selected because of the extensive nature of the research. The researchers were encouraged by Christ (2018), who suggests that a mixed methods approach is feasible in research concerning special education or education of people with disabilities. Qualitative data were collected through reviewing literature, conducting document reviews, as well as semistructured interviews with information-rich groups of participants. Quantitative data were collected through analysis of attendance registers of registered students and Kirkpatrick Level 1 Student Satisfaction feedback forms.

The problem of devising an appropriate research method was complex, as it involved the Training Department, students, alumni and employers. A case study using a mixed methods approach was selected to investigate how students could be trained and helped to find and keep employment in disability-sensitised work environments (Corrigan & Onwuegbuzie 2020). The research paradigm was interpretive, and purposeful sampling allowed for information-rich participants (Rout 2019). There were elements of action research in the investigation; that is, after an investigation of needs, certain actions are planned and implemented in cycles to determine success. Cycles consist of action (or involvement), evaluation of and reflection on results, repeated cycles in which some elements are changed, results are assessed, some more changes are made and the process is repeated. However, the method used in this research did not repeat similar processes. Christ (2018) claims that mixed methods research and action research are comparable because both can use qualitative as well as quantitative data in one study.

The research took place in five phases. The first phase was qualitative and included a review of documents and a literature review, in addition to semistructured interviews. Six small groups participated, namely students studying on-campus, students studying off-campus but enrolled at the institution, alumni who had been part of previous work placement initiatives, facilitators, support staff and employers of alumni.

The two streams of literature review provided information and underpinned the mixed methods used in the research. The questions asked in semistructured interviews stemmed from the literature study, while the document review of various government Acts, regulations and codes of best practice, minutes of meetings and other official documents, led to the framework used to analyse the training models. Both types of literature study indicated the need to include the views of students; thus, in the second phase, two quantitative methods were applied, namely a student satisfaction questionnaire and the analysis of student attendance (Corrigan & Onwuegbuzie 2020).

As no suitable assessment instrument was available, it was necessary to develop one that was fit for purpose. An assessment instrument or model, namely the Adaptable Component-based Assessment Model (ACA Model), was developed by the researchers to provide consistent analyses of training models at the institution. As the name suggests, it is adaptable so that it can be used in other training environments. This article reports on the ACA Model that was developed.

### First phase of research

Each component of the ACA Model has students as its focus. The relevance to job creation, student satisfaction and class attendance are important because they indicate how future-focused the programme is and also whether students feel they are benefitting from the educational opportunity (Dennis et al. 2016). The rest of the components indicate the institution’s responsibilities to ensure the success of the programme.

The ACA Model is structured in the form of a matrix with four vertical and eight horizontal axes. The content of each of the intersecting blocks is selected so that individual elements can be assessed. The list of model descriptors is comprehensive but not prescriptive, and components that meet the needs of an individual institution can be added.

The core components are identified according to the programme and the environment in which it is offered. The selection of the components must be considered with care to ensure that only those that are essential are selected, and they must be used consistently during an analysis. Examples of core components are meta-competencies, cognitive abilities and methodological knowledge, functional and technical competencies, personal competence, values and ethics competence, individual affordances and workplace affordances, holistic development, the context of the workplace and reflective learning. The competencies reflected in the model enable an assessor to evaluate a programme from the point of view of a training organisation, students and prospective employers.

Meta-competencies are seen as relevant, overarching competencies like adapting, anticipating, learning and creating changes that generate flexibility in various work environments (Heery & Noon 2017). Cognitive competencies include the ability to apply knowledge and skills in real-life situations, and methodological knowledge refers to theoretical knowledge of learnt skills and their methods. Functional competencies are often technical or operational in nature and reflect the competencies required to perform a task effectively (Garwe 2020). On the other hand, essential skills for building respectful, diverse and inclusive workplaces where employers hold themselves and their employees accountable for their actions are more personal than organisational. Personal competence refers to emotional intelligence and the ability individuals have to manage their lives and emotions. Social competence is behavioural in nature and refers to self-regulation, positive self-identity and social adaptation, while values and ethics competency means that personal and organisational practices are performed with integrity and respect (Vornholt et al. 2018). The individual affordances included in the model are those that regulate human behaviour and are formed between an individual and an environment. Holistic development includes physical, social, emotional, mind and spiritual learning and growth.

Workplace affordances focus on whether and how employers provide opportunities for learning. It is important to incorporate the context of the workplace because it includes awareness, acceptance, respect and understanding in an environment where everyone is valued for their unique skills, experiences and perspectives (Vornholt et al. 2018). Reflective learning is an intentional process in which students make meaning of the learning experience and think about what they have learned (Brockbank et al. 2017). This helps them to develop critical and creative thinking skills and encourage active engagement in learning (Brockbank et al. 2017). As can be seen from above, the core competencies evaluate the learning experience from a 360° perspective.

Table 1 provides the basic components of the ACA Model as a matrix. These components are not obligatory, as only those that are applicable need to be included in an assessment.

**TABLE 1:** Basic components of the Adaptable Component-based Assessment Model.

Model descriptors	Core components (Selected by institution)	Core competencies	Success indicators (Selected by institution)
Programmes offered and relevance to job creation	Mode of training delivery	Meta-competencies	-
Number of learners and students enrolled	Mode of operation	Cognitive competence methodological knowledge	-
Academic (facilitator) and support staff to student ratio	Mode of funding	Functional and technical competence	-
Theoretical and practical components of programmes	Risks and mitigating actions	Personal competence	-
Special needs of students	Disability catered for	Values and ethics competence	-
Recruitment of students	Mode of awareness-making	Holistic development	-
Funding requirements	Accommodations	Workplace affordances	-
	Accommodations	Individual affordances	-
Core components of the training model	Curriculum	Reflective learning	-

*Source:* Janse van Rensburg-Welling, J.C., 2020, ‘Accessible career paths for students with different degrees of hearing loss at the National Institute for the Deaf’, PhD thesis, Da Vinci Institute of Technology Management.

### Adaptability of the Adaptable Component-based Assessment Model

The adaptability of the ACA Model allows an organisation to select the most appropriate model descriptors, core components, core competencies and success indicators once it has defined their own scope of work as well as the aims and objectives of the programme. The model descriptors should be as extensive as possible and must include the aspects that will allow the organisation to reach the goals of the programme when implementing its envisioned strategies.

The core components focus on ‘unpacking’ the model descriptors to describe the most essential elements. In the research reported here, the mode of training delivery was identified as the most crucial core component, as it deals directly with the training and workplace preparation of students for the rapidly changing world of work (Keengwe & Byamukama 2019). Mode of operation, mode of funding and identified risks and mitigating actions were also regarded as core components. The competencies expected of students when they have completed a programme flow from the model descriptors and core components.

As the programmes offered by the organisation where the research was conducted focused on work-related practice, the evaluation instrument included meta-competencies, cognitive abilities and methodological knowledge, functional, technical and personal competencies, values and ethics, individual and workplace affordances, as well as development and reflective learning to analyse the mode of delivery. Success indicators that were selected in the research have not been included here, but suffice it to say that assessing success in any programme must reflect the aims and objectives identified at the beginning of the assessment process. As each element is assessed, it is inevitable that more elements will be added. Thus, the assessment matrix can become flexible and even elastic. In this way, it is made fit for purpose for individual organisations.

In the present research, the ACA Model was first applied to the original training model of the organisation. As a result of the assessment, certain changes were made and applied in programmes the following semester. The adapted programme was then assessed using the same elements to ensure consistency and to assess which of the adaptations were successful and which were not. The two assessments are presented in Table 2. As this is an example, only the expanded elements of mode of training delivery are shown. It is evident from the matrix that once the elements are decided upon, the actual assessment is fairly uncomplicated. As in the example below, if the same elements are used to assess an adapted programme, successes and failures become clear.

**TABLE 2:** Application of Adaptable Component-based Assessment Model.

Elements of mode of training delivery	Applied in original training model	Applied in adapted programme
Meta-competencies	No, because of limited exposure	Yes, because of broader workplace exposure
Cognitive abilities and methodological knowledge	No, because of limited exposure	Yes, because of broader workplace exposure and real-life situations
Functional and technical competencies	No, because of limited exposure	Yes, because of rigorous recruitment process
Personal competence	Yes, because of programmes being offered by ISS	Yes, because of programmes being offered by HR teams of sponsoring companies
Values and ethics competency	Yes, because of induction and code of conduct programmes being offered	Yes, because of programmes being offered by HR teams of sponsoring companies
Individual affordances	No, because of limited exposure	Yes, because of broader workplace exposure
Holistic development	No, because of limited exposure	Yes, because of broader workplace exposure
Inclusion of context at the workplace	No, because of limited exposure	Yes, because of broader workplace exposure
Reflective learning	No, because of limited exposure	Yes, because of broader workplace exposure

*Source:* Janse van Rensburg-Welling, J.C., 2020, ‘Accessible career paths for students with different degrees of hearing loss at the National Institute for the Deaf’, PhD thesis, Da Vinci Institute of Technology Management.

ISS, integrated support services; HR, human resources.

## Conclusion

Training programmes for persons with disabilities should be future focused and provide choices at different National Qualifications Framework (NQF) levels so that appropriate career choices can be made available. Various training models can be used to train students with disabilities, but they must be integrated, holistic and student centred. In order to achieve and maintain these standards, programmes need to be evaluated at the outset and at regular intervals. Thus, an adaptable, structured assessment tool, namely the ACA Model, has potential to facilitate such programme evaluations. The ACA training assessment model includes predetermined project aims and objectives, resources, roles and responsibilities of role players, as well as a cycle of programme assessments and adaptations. It enables ongoing evaluation of individual programmes, as well as complete courses. Using the ACA assessment model can potentially add value to the development of all programmes.
